# Supplementary figures and images for: Homophily and the Speed of Social Mobilization: The Effect of Acquired and Ascribed Traits
Source: PLoS One. 2014 Apr 16;9(4):e95140. doi: 10.1371/journal.pone.0095140 (PMC3989266; doi:10.1371/journal.pone.0095140)

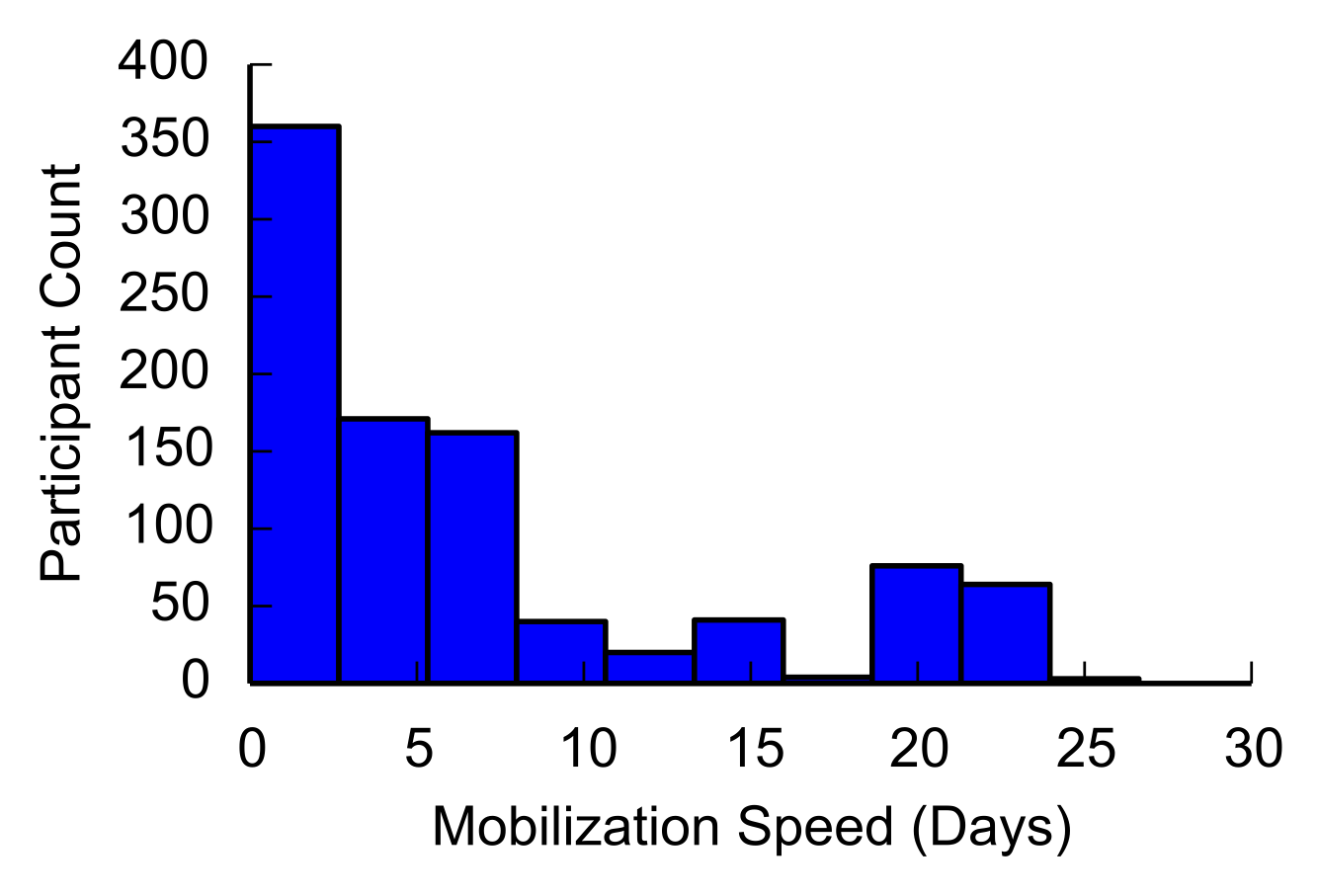

Supplement: Figure S1 — The distribution of mobilization speeds was heavy-tailed. Mobilization speeds were measured by the interval between when a recruiter registered on the contest website and when their recruit registered. The mean mobilization speed was 6.7 days, with a standard deviation of 7.2 days. (TIFF) [file pone.0095140.s001.tiff]

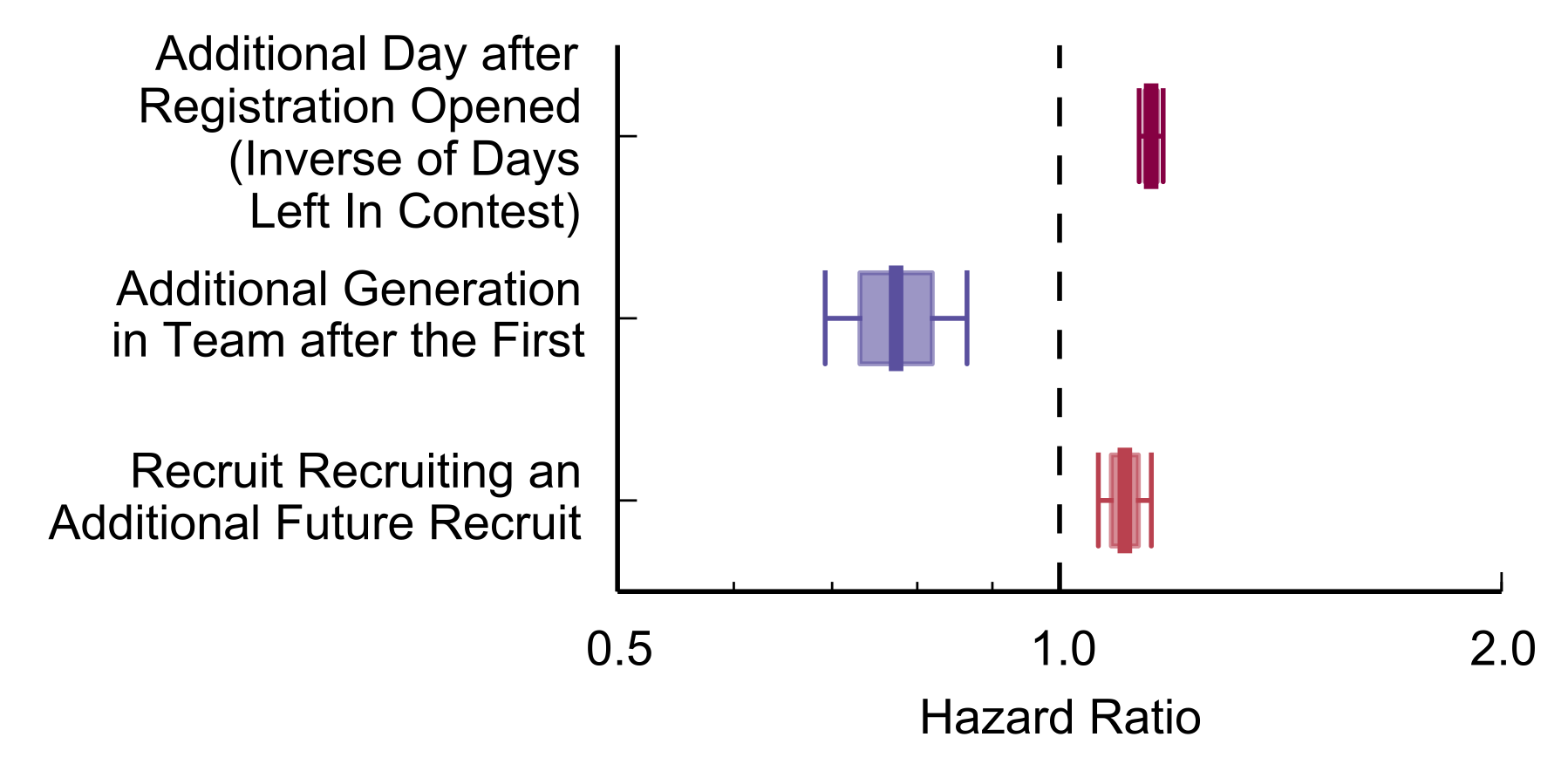

Supplement: Figure S2 — Time left in the contest, additional generations, and additional future recruits all affected mobilization speed. The further in time the recruiting happened (i.e. closer to the contest date), the faster the mobilization speed. In contrast, as a team grew with generations of recruiters recruiting recruits, each additional generation beyond the first (hazard ratio = 1) slowed down mobilization speed. The recruit's mobilization speed increased for each additional future recruit he or she had beyond zero. (TIFF) [file pone.0095140.s002.tiff]
