# Supplementary material for: Homophily and the Speed of Social Mobilization: The Effect of Acquired and Ascribed Traits
Source: PLoS One. 2014 Apr 16;9(4):e95140. doi: 10.1371/journal.pone.0095140 (PMC3989266; doi:10.1371/journal.pone.0095140)
Supplement: Information S1 — Goodness of fit measures for the Cox proportional hazards model. (PDF) [file pone.0095140.s003.pdf]

Supporting Information for

Measuring and Predicting Speed of Social Mobilization

Goodness of Fit

We calculated several metrics for the goodness of fit of the Cox proportional hazard model. The model had 44 degrees of freedom. All tests showed that the model was a better fit to the data than a null model

Metric	*Value*
Likelihood ratio test	998.2 ($p<.0001$)
Wald test	890.6 ($p<.0001$)
Score test	1108 ($p<.0001$)

Additionally, we calculated the concordance probability of the model, which compared the relative order of all pairs of participants in the empirical data versus the model. The concordance was 80.8%, a good probability.

Proportional Hazards Assumption Testing

The proportional hazards model makes the assumption that the hazard functions of different factor values are proportional across time. To check for violations to this assumption, we plotted the scaled Schoenfield residuals for all explanatory variables (below). The lack of linear trends for any of these residuals indicates that the proportional hazards assumption holds.

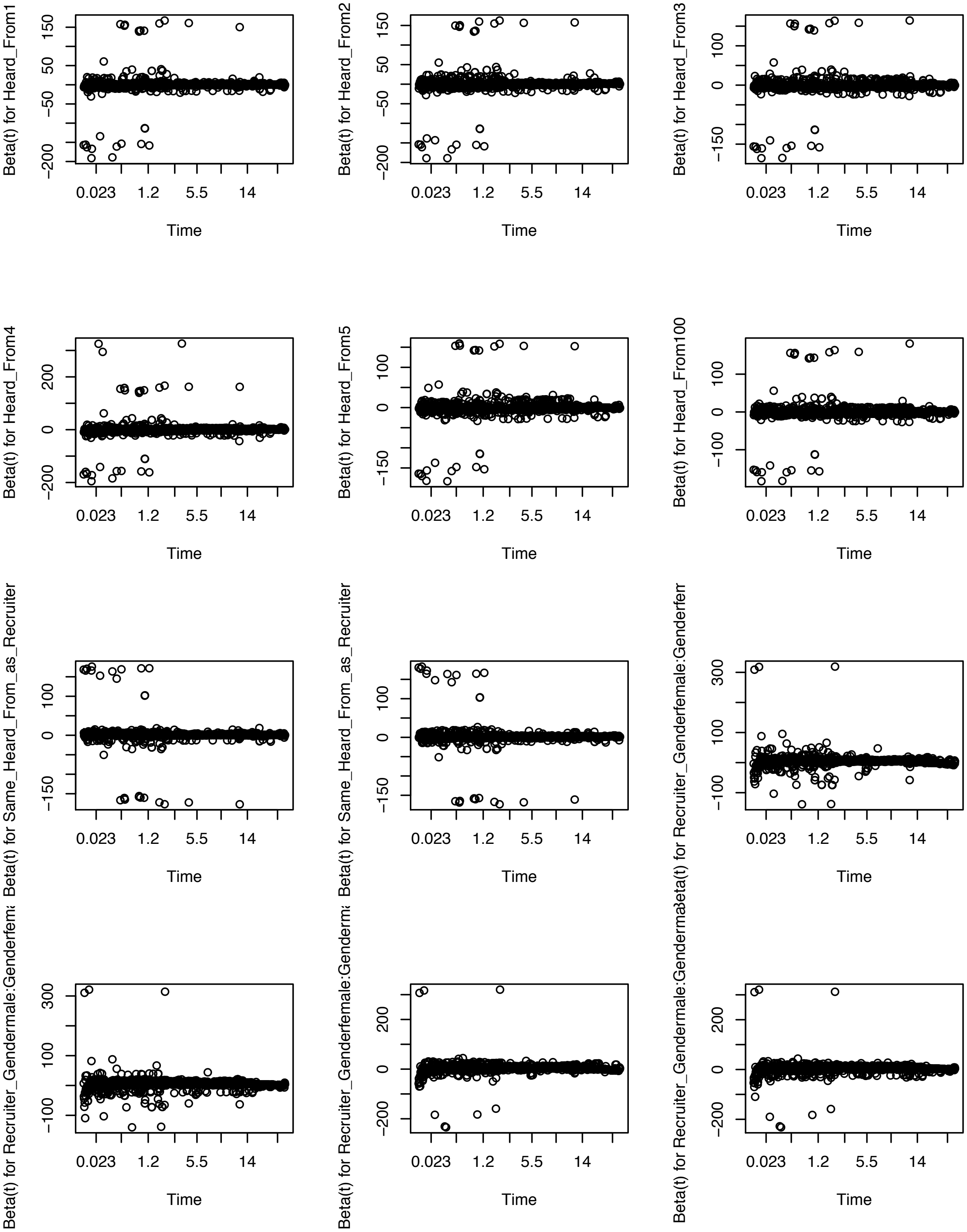
Beta(t) for Heard_From1
Beta(t) for Heard_From2
Beta(t) for Heard_From3
Beta(t) for Heard_From4
Beta(t) for Heard_From5
Beta(t) for Heard_From100
Beta(t) for Same_Heard_From_as_Recruiter
Beta(t) for Same_Heard_From_as_Recruiter
Beta(t) for Recruiter_Genderfemale:Genderferr
Beta(t) for Recruiter_Gendermale:Genderfem:
Beta(t) for Recruiter_Genderfemale:Genderm:
Beta(t) for Recruiter_Gendermale:Genderma
0.023
1.2
5.5
14
Time

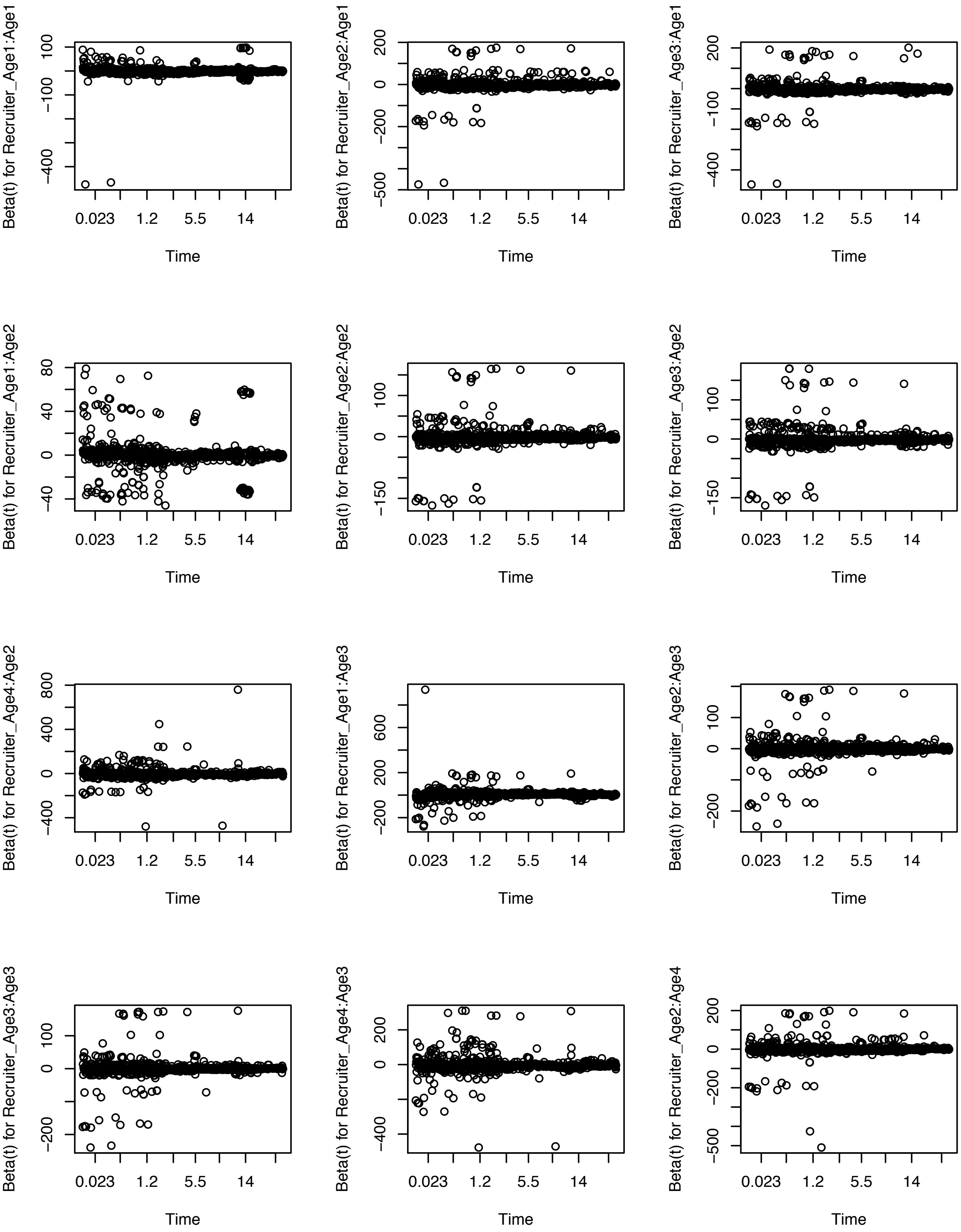

Beta(t) for Recruiter_Age1:Age1
100
−100
−400
Beta(t) for Recruiter_Age2:Age1
200
0
−200
−500
Beta(t) for Recruiter_Age3:Age1
200
−100
−400
Beta(t) for Recruiter_Age1:Age2
80
40
0
−40
Beta(t) for Recruiter_Age2:Age2
100
0
−150
Beta(t) for Recruiter_Age3:Age2
100
0
−150
Beta(t) for Recruiter_Age4:Age2
800
400
0
−400
Beta(t) for Recruiter_Age1:Age3
600
200
−200
Beta(t) for Recruiter_Age2:Age3
100
0
−200
Beta(t) for Recruiter_Age3:Age3
100
0
−200
Beta(t) for Recruiter_Age4:Age3
200
0
−400
Beta(t) for Recruiter_Age2:Age4
200
0
−200
−500
0.023
1.2
5.5
14
Time

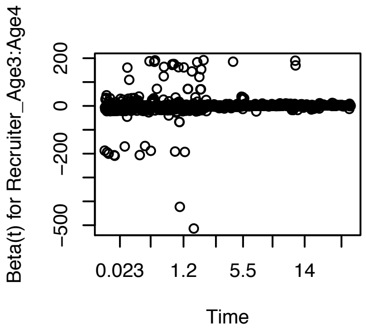

Beta(t) for Recruiter_Age3:Age4
200
0
−200
−500
0.023
1.2
5.5
14
Time
